# Psychosomatic Features of Irritable Bowel Syndrome: The Role of Alexithymia in Patient Health-Related Quality of Life—A Cross-Sectional Study

**DOI:** 10.3390/healthcare14050562

**Published:** 2026-02-24

**Authors:** Emanuele Maria Merlo, Liam Alexander MacKenzie Myles, Angela Alibrandi

**Affiliations:** 1 Department of Biomedical and Dental Sciences and Morphofunctional Imaging, University of Messina, 98124 Messina, Italy; 2 Berkshire Healthcare NHS Foundation Trust, Bracknell RG12 2UT, UK; liam.a.myles@outlook.com; 3 Department of Human Pathology in Adulthood and Childhood Gaetano Barresi, University of Messina, 98124 Messina, Italy; aalibrandi@unime.it

**Keywords:** alexithymia, clinical psychology, health status, IBS, irritable bowel syndrome, psychosomatic disorders, psychosomatics

## Abstract

*Background*: Psychological factors such as alexithymia, somatization, and their associated effects on health are consistently observed as key characteristics in the onset, maintenance, and chronic course of irritable bowel syndrome (IBS). This study aimed to investigate the presence and role of alexithymia, psychosomatic syndromes, and health status in individuals affected by IBS. *Methods*: The sample comprised 143 patients diagnosed with IBS, ranging in age from 18 to 78 years (M = 30.90, SD = 14.39), with a predominance of females (81.8%). The diagnostic protocol included a sociodemographic questionnaire, the Toronto Alexithymia Scale-20 (TAS-20), the Diagnostic Criteria for Psychosomatic Research-Structured Interview (DPCR-SI), and the SF-36 Health Survey. Descriptive, correlational, and generalised regression analyses were conducted to examine the relationships among these variables. *Results*: The findings revealed significant positive correlations between alexithymia and psychosomatic syndromes, while significant negative correlations were observed with health status. Furthermore, alexithymia was identified as a predictor of increased psychosomatic syndrome severity and reduced health status. *Conclusions*: These results highlight the critical role of alexithymia in IBS and underscore the importance of developing targeted interventions to address this psychological factor in affected individuals.

## 1. Introduction

Irritable Bowel Syndrome (IBS) is one of the most common functional gastrointestinal disorders, producing substantial implications for patients’ Health-Related Quality of Life (HR-QoL) [1]. It represents a chronic gastrointestinal disorder mainly characterized by altered bowel habits, evacuation frequency and feces consistency, discomfort and abdominal pain [2,3]. The diagnosis is complex as symptoms can change during lifespan and be attributable to other syndromes [4,5,6]. According to Oka and colleagues [1], IBS affects between 1 in 11 and 1 in 26 people globally. According to a recent meta-analysis [7], the pooled estimated prevalence of IBS among Italian adults is approximately 7.7%, although rates vary depending on diagnostic criteria and assessment methods. This figure reflects epidemiological estimates reported in the literature rather than data derived from the present sample.

Recent research has increasingly focused on the psychological functioning of patients, particularly concerning the consequences associated with physical manifestations, the etiological contribution of psychological disturbances, and their role in the maintenance of pathological conditions [8,9,10].

In particular, functional gastrointestinal disorders, including irritable bowel syndrome (IBS), are increasingly conceptualized within a biopsychosocial framework in which psychological distress and stress-related processes play a central role in symptom onset, exacerbation, and illness course [11,12]. Inflammatory bowel diseases likewise show high rates of anxiety and depressive symptoms, further supporting the relevance of psychological dimensions in gastrointestinal conditions [8,9]. Emerging evidence also highlights the contribution of specific affective processes, such as disgust sensitivity and maladaptive coping, to symptom perception and adjustment in IBS and related disorders [10,13].

Beyond gastrointestinal conditions, alexithymia has been consistently identified as a clinically relevant factor across several chronic medical illnesses. A recent systematic review further supports the relevance of alexithymia in metabolic and autoimmune conditions, consolidating its transdiagnostic significance in chronic disease contexts [14].

The psychogenic contribution of these factors provides insight into patients’ overall burden and health-related limitations [15,16,17]. Moreover, psychological factors are now widely recognized as causal mechanisms and risk factors for IBS [18,19,20], significantly influencing its diagnosis and course.

Recent studies have emphasized the significant impact of affective dynamics and alterations on patients’ functioning in the context of IBS, often contributing to the development of psychosomatic disorders [21,22,23,24]. Several specific psychological phenomena are of particular relevance in this regard.

Alexithymia can be defined as the inability to recognize, identify and describe affective manifestations such as emotions, feelings and mood states [25,26,27]. A widely accepted theoretical model conceptualises alexithymia as comprising three core dimensions: difficulty identifying feelings (DIF), difficulty describing feelings (DDF), and a tendency toward externally oriented thinking (EOT) [26].

The link between alexithymia and physical illness results of fundamental interest [28,29]. Its pathogenic role has been recognized by several studies referring to a wide range of disorders [30,31,32,33,34,35,36]. Chronic diseases appeared to be strongly associated with alexithymia [37,38,39]. The relationship between alexithymia and gastrointestinal disorders appears to be even clearer considering recent and past studies [40,41].

According to Boudabbous and colleagues [41], alexithymia impairs HR-QoL of people suffering from IBS. Highlighting the limited understanding of IBS etiopathogenesis, the authors observed a high prevalence of alexithymia, which was associated with reduced HR-QoL. In addition to psychological factors, IBS was found to impose concrete limitations on daily activities, including behavioural and dietary restrictions, impaired HR-QoL, and diminished perceptions of health status [42,43,44].

A recent study by Trindade and colleagues [45] further explored this issue, reporting lower HR-QoL among IBS patients, a higher prevalence among females, and the direct impact of IBS on mental and physical well-being. Their findings also highlighted the significant influence of somatic symptoms on physical HR-QoL. Based on these results, the authors emphasized the urgent need for targeted psychological interventions.

Consistent with these findings, Cassar and colleagues [46], in a systematic review and meta-analysis, confirmed the high global prevalence of IBS, affecting up to 20% of the population, and highlighted the substantially poorer HR-QoL experienced by affected individuals. Notably, those with the lowest HR-QoL represented a broader segment of the IBS population. The authors underscored the importance of further research addressing the psychological functioning and HR-QoL of individuals with IBS, emphasizing the need for more comprehensive and integrative approaches.

### Study Hypotheses

Based on previous evidence highlighting the role of affective processing deficits in health-related outcomes, the following hypotheses were formulated:

**H1.**  *Increasing age is associated with lower health status, and variable effects on psychosomatic symptoms, whereas higher education is expected to be associated with lower alexithymia.*

**H2.**  *Higher levels of alexithymia are expected to be associated with poorer health-related quality of life and increased psychosomatic symptomatology.*

**H3.**  *Higher levels of alexithymia are expected to show dependencies with lower health status and greater psychosomatic symptom severity.*

**H4.**  *Gender differences are expected in levels of alexithymia and health-related quality of life, with men and women differing on these variables.*

## 2. Materials and Methods

### 2.1. Participants

The study involved 143 patients suffering from irritable bowel syndrome (exacerbation phase) aged between 18 and 78 years old (M: 30.90; SD: 14.39) with a female prevalence of 81.8%. For this cross-sectional clinical research, all people were sent by General Practice reporting an IBS diagnosis and recruited at the University Hospital “Gaetano Martino” of Messina, Messina, Italy, during the normal clinical activities starting from January 2020 to September 2023. Inclusion criteria were diagnosis of IBS, absence of comorbidity, absence of major psychopathological disorders or medications influencing cognitive functioning. Assuming a prevalence of irritable bowel syndrome in the general population of 0.385% (38.5 per 10,000 subjects) [15] and an expected prevalence of 3% in the study group (motivated by the fact that recruitment takes place in a University Hospital), with a significance level of 5% and a minimum power of 80%, the required sample size was calculated to be at least 103 patients. All recruited patients agreed to participate in the study and signed informed consent. All patients fully completed the diagnostic protocol. The present study and all related procedures were consistent with the Declaration of Helsinki (1964) and its amendments. The study was approved by the Ethical Committee of the University Hospital “Gaetano Martino” of Messina, Messina, Italy (Approval number: 40/19, 3 July 2019).

### 2.2. Instruments

The study protocol consisted of a clinical interview, a sociodemographic questionnaire, and validated instruments for assessing psychological variables. The sociodemographic variables collected were age, gender, and educational level.

#### 2.2.1. Toronto Alexithymia Scale (TAS-20)

The Toronto Alexithymia Scale (TAS-20) [47] is a self-report scale composed of 20 items based on a 5-point Likert scale ranging from “strongly disagree” to “Strongly agree” assessing alexithymia. The scale is mainly composed of three factors: difficulty identifying feelings (DIF), difficulty describing feelings (DDF) and externally oriented thinking (EOT). It represents a widely recognized valid instrument for the assessment of alexithymia and related factors in a wide set of pathological and non-pathological domains. The original validation study demonstrated its internal consistency. Cronbach’s alpha indexes were: 0.81 for full scale; 0.78 for DIF; 0.75 for DDF; 0.66 for EOT. The Italian adaptation of the scale [48] showed good internal consistency: 0.82 for the full scale, 0.79 for DIF, 0.68 for DDF and 0.54 for EOT.

#### 2.2.2. SF-36 (Short Form-36)

The Short Form Health Survey (SF-36) is a 36-item scale assessing the health status (Italian version by Apolone & Mosconi [49]; Hays & Sherbourne [50], RAND Medical Outcomes Study). According to the validation study, the various factors reached the following alphas: Physical functioning 0.93, Role limitations due to physical health 0.84, Role limitations due to emotional problems 0.83, Energy/fatigue 0.86, Emotional well-being 0.90, Social functioning 0.85, Pain 0.78, General health, 0.78. Regarding its internal consistency a IQOLA projects involving the Italian version confirmed its validity [51,52,53] (ranging from 0.68 to 0.94).

#### 2.2.3. Diagnostic Criteria for Psychosomatic Research (DCPR)

The Diagnostic Criteria for Psychosomatic Research Structured Interview (DCPR-SI) [54] is a structured interview including a set of 12 syndromes. The scales are disease phobia, thanatophobia, health anxiety, illness denial, persistent somatization, functional somatic symptoms secondary to a psychiatric disorder, conversion symptoms, anniversary reaction, irritable mood, type A behavior, demoralization, and alexithymia. Galeazzi and colleagues [55] reported high interrater reliability with the following Kappa values: Disease phobia, 0.97; Thanatophobia, 0.92; Type A behavior, 0.92; Illness denial, 0.90; Demoralization, 0.90; Anniversary reaction, 0.90; Health anxiety, 0.89; Alexithymia, 0.89; Conversion symptoms, 0.82, Persistent somatization, 0.70; and Irritable mood, 0.69.

### 2.3. Statistical Analysis

Categorical variables were expressed as numbers and percentages, and numerical data through means and standard deviation. The Kolmogorov–Smirnov test was used to assess the normality of the distribution. After detecting the non-normality for some of the distributions, a non-parametric approach was adopted. Correlation analyses were performed using the Spearman test. Generalised linear regressions (GLMs) were performed to study the dependence of clinical variables on alexithymia. This approach was chosen to account for the non-normal distribution of some of the variables. These models complement the non-parametric correlation analyses by allowing adjustment for covariates. The Mann–Whitney test was used to analyse differences in alexithymia and quality of life between male and female participants. Correlations among covariates were examined using Spearman’s rank correlation coefficients. To assess multicollinearity in the GLMs, variance inflation factors (VIFs) were calculated for all predictors. All VIFs were below 2.5, indicating no problematic multicollinearity, and therefore all covariates were retained in the models. Given that each hypothesis was considered separately and analyses were exploratory in nature, no formal adjustment for multiple comparisons was applied. Results are interpreted with caution, acknowledging the potential for type I error. The *p*-value < 0.05 was significant. Statistical analyses were performed using SPSS 25 for Windows.

## 3. Results

Descriptive statistics, correlations and regressions are reported (see Table 1).

Descriptive statistics revealed the prevalence of clinical variables within the sample. The overall level of alexithymia had a mean score of 55.05, which falls within the borderline range. Regarding psychosomatic syndromes, there was a high prevalence of persistent somatization, along with notable occurrences of health anxiety, illness denial, Type A behavior, and demoralization.

Table 2 presents the results of the correlational analysis conducted with age, education, alexithymia, health status, and psychosomatic variables. Regarding age, significant positive correlations were observed with EOT, role limitations due to emotional problems, energy/fatigue, and emotional well-being, indicating that higher age was associated with increased scores on these variables. Conversely, significant negative correlations were identified with DIF, physical functioning, general health, Type A behaviour, and demoralization, suggesting that advancing age was associated with lower scores on these scales.

Concerning education, significant negative correlations were found with DIF and DDF. These results indicate that higher levels of education were associated with lower scores on these two dimensions of alexithymia.

Table 3 presents the results of the correlational analysis among alexithymia factors, health status variables, and psychosomatic syndromes. For the total score on the Toronto Alexithymia Scale (TAS-20), significant positive correlations were found with health anxiety, disease phobia, thanatophobia, persistent somatization, Type A behavior, demoralization, and general alexithymia. These results indicate that higher levels of alexithymia are associated with an increased presence of psychosomatic phenomena. Conversely, significant negative correlations were observed with physical functioning, role limitations due to emotional problems, emotional well-being, social functioning, pain, and general health, suggesting that elevated alexithymia levels are linked to diminished health status. Thus, higher alexithymia levels were consistently associated with lower health status and a greater prevalence of psychosomatic symptoms.

This pattern was particularly evident in the TAS-20 subscales measuring DIF and DDF. Higher levels of these subscales were associated with reduced health status and greater psychosomatic symptomatology. Specifically, significant negative correlations were observed between DIF and variables such as role limitations due to physical and emotional health, emotional well-being, social functioning, and pain. In contrast, significant positive correlations emerged between DIF and disease phobia, thanatophobia, Type A behavior, irritable mood, and demoralization.

For the DDF subscale, significant negative correlations were identified with physical functioning, role limitations due to emotional problems, social functioning, pain, and general health. Positive and significant correlations were found with disease phobia, Type A behavior, and overall alexithymia.

The EOT subscale showed significant associations, with increasing levels corresponding to lower physical functioning and higher levels of energy/fatigue and thanatophobia.

Table 4 presents the results of the correlational analysis between health status and psychosomatic syndrome variables. Higher levels of physical functioning were associated with lower levels of thanatophobia and illness denial.

Increased role limitations due to physical health were positively correlated with persistent somatization and negatively correlated with Type A behaviour, irritable mood, and demoralization. All significant correlations involving role limitations due to emotional health were negative and included conversion symptoms, Type A behaviour, irritable mood, and demoralization.

Higher energy/fatigue scores were positively correlated with conversion symptoms and negatively correlated with Type A behaviour, irritable mood, and demoralization. Similarly, significant correlations with emotional well-being were negative, encompassing thanatophobia, Type A behaviour, irritable mood, and demoralization.

Social functioning was positively correlated with persistent somatization and negatively correlated with illness denial, Type A behaviour, irritable mood, and demoralization. Pain demonstrated significant positive correlations with persistent somatization and significant negative correlations with illness denial and irritable mood.

Negative correlations were observed between general health and psychosomatic syndromes, including disease phobia, thanatophobia, functional somatic symptoms secondary to psychiatric disorders, irritable mood, and demoralization.

A noteworthy pattern emerged—while all significant correlations between health status and psychosomatic syndromes were negative, the correlations involving persistent somatization were consistently positive. This finding warrants further discussion and comparison with the existing literature to better understand its implications.

Table 5 presents the results of the generalised regression analysis, with the total score on the Toronto Alexithymia Scale (TAS-20) as the predictor and health status and psychosomatic variables as outcome measures. Consistent with the findings from the correlational analysis, alexithymia was identified as a significant predictor of reduced health status and elevated psychosomatic symptomatology.

Specifically, higher levels of alexithymia were associated with lower scores on health status variables, including physical functioning, role limitations due to emotional problems, emotional well-being, social functioning, pain, and general health. In terms of psychosomatic conditions, alexithymia was associated with higher levels of disease phobia, thanatophobia, Type A behaviour, irritable mood, demoralization, and clinically assessed alexithymia.

Table 6 presents the results of generalised regression analyses examining the relationships between the factors of the Toronto Alexithymia Scale (TAS-20) as predictors and health status and psychosomatic variables as outcomes. Consistent with the findings from Table 5, the significant relationships identified followed the same patterns as the correlations. Specifically, all significant associations between DIF and DDF and health status were negative, indicating a deterioration in health associated with alexithymia. In contrast, all significant associations between these alexithymia factors and psychosomatic syndromes were positive, highlighting the role of alexithymia in the onset and maintenance of psychosomatic disorders.

Significant negative associations involving DIF were observed with role limitations due to emotional problems, emotional well-being, social functioning, pain, and general health. Positive associations emerged with disease phobia, thanatophobia, illness denial, conversion symptoms, Type A behaviour, irritable mood, and demoralization. A significant negative association was found between DDF and role limitations due to emotional problems, while significant negative associations were identified with conversion, Type A behaviour and clinically detected alexithymia.

EOT was found associated with higher levels of role limitations due to emotional problems, energy/fatigue, and emotional well-being. Conversely, lower levels of Type A behaviour, irritable mood, and demoralization emerged.

Table 7 presents the results of the gender comparison analyses. No significant differences were observed between male and female patients for alexithymia (TAS-20) or health-related quality of life (SF-36) scores.

## 4. Discussion

The study revealed meaningful relationships between alexithymia, health status, and psychosomatic symptoms. These findings are particularly relevant considering the neurobiological pathways implicated in IBS, suggesting that difficulties in emotional awareness and regulation may translate into somatic symptom amplification via dysregulated gut–brain axis communication, HPA axis hyperactivity, and autonomic imbalance.

Irritable Bowel Syndrome (IBS) is a multifactorial disorder in which factors interact to shape symptom expression and patient experience. Understanding the interplay between emotional processing and physiological mechanisms is therefore essential to elucidate the pathophysiology of IBS as a disorder of gut–brain interaction [56,57].

A compelling body of evidence supports neurobiological mechanisms linking alexithymia, emotion dysregulation, and somatization in IBS through dysregulated gut–brain axis communication. IBS is increasingly recognised as a disorder of gut–brain interaction in which altered bidirectional signalling between the enteric nervous system, central nervous system, and neuroendocrine pathways contributes to visceral hypersensitivity, abnormal motility, and stress responsivity [58,59]. Difficulty identifying and describing emotions may amplify somatic symptom perception by interfering with effective stress regulation, such that emotional arousal chronically engages stress response systems, including the HPA axis and the autonomic nervous system [60]. In patients with IBS, higher alexithymia scores have been associated with stronger endocrine stress responses (e.g., adrenocorticotropic hormone levels) following corticotropin-releasing hormone challenge, supporting a link between emotional processing difficulties and HPA axis engagement [61]. Chronic stress and sustained HPA activation can lead to elevated cortisol and catecholamines, which influence gut motility, intestinal permeability, and visceral sensitivity, all of which are key pathophysiological features of IBS [62,63]. This mechanism suggests that individuals with high alexithymia may experience enhanced sympathetic nervous system activity and reduced vagal tone, leading to heightened visceral pain perception and somatic amplification. Indeed, altered central processing of visceral stimuli has been reported in individuals with higher alexithymia, indicating that emotional awareness deficits may modulate visceral hypersensitivity and maladaptive gut–brain signalling in IBS [59,61]. Meta-analytic work has confirmed elevated levels of alexithymia in IBS relative to controls, indicating that these emotional processing difficulties are more prevalent in IBS and may contribute to symptom severity and psychological burden [64]. Together, these findings support a model in which dysregulated emotion processing interacts with neuroendocrine and autonomic dysregulation to convert emotional distress into somatic symptom amplification in IBS.

Through the analyses conducted, all four hypotheses were examined by exploring the relationships between alexithymia, quality of life, and psychosomatic variables.

Regarding the first hypothesis, several significant correlations emerged between age, alexithymia, health status, and psychosomatic syndromes. Notably, positive and significant correlations indicated that age was associated with increased levels of EOT, role limitations due to emotional problems, energy/fatigue, and emotional well-being. The continuity of EOT with ageing has been well-documented in previous research. Schooler and colleagues [65], as confirmed by Irish et al. [66], emphasized the relevance of this phenomenon and its relationship with internally focused attention. However, any interpretation of results pertaining to this subscale must be approached with caution, as the internal consistency may limit the robustness of conclusions drawn from these data.

The high prevalence of alexithymia in this sample is consistent with previous studies. Increased age was associated with higher levels of emotional limitations, energy/fatigue, and emotional well-being. A study by Tang et al. [67] on age-related changes and HR-QoL in individuals with IBS found that younger individuals experienced more emotional problems and related energy/fatigue issues, supporting the positive correlations identified in the first hypothesis.

Conversely, negative and significant correlations were found between age and DIF, physical functioning, health anxiety, Type A behavior, and demoralization. Ageing was associated with reduced levels of alexithymia expression, worse general health status, and lower levels of Type A behavior and demoralization. While alexithymia is generally considered to vary across different phases of the lifespan [68], some studies have indicated higher levels in older populations [69]. In the general population, alexithymia has been strongly associated with ageing, particularly neurocognitive deficits that influence emotional and cognitive processing, a concept known as the deficit hypothesis.

In our study, the analysis revealed an inverse relationship between alexithymia and ageing. This intriguing finding warrants further investigation and the development of additional hypotheses.

The inverse association between age and alexithymia constitutes one of the most theoretically informative findings of the present study. Rather than contradicting the deficit hypothesis, this result invites a more developmentally refined interpretation of the model. The deficit framework conceptualises alexithymia as reflecting relatively stable individual differences in emotional awareness and affect regulation, without positing a necessary linear increase across the adult lifespan [70]. Empirical findings on age-related trends remain heterogeneous. While some cross-sectional investigations have reported higher alexithymia scores in older cohorts [68], subsequent research suggests that such patterns may be influenced by cohort effects, sociocultural norms regarding emotional expression, or educational differences rather than by age-related structural decline per se [71]. Moreover, lifespan perspectives emphasise that emotional regulation capacities and motivational selectivity may strengthen with age, potentially facilitating improved emotional integration in adulthood [72]. Recent evidence further indicates that distinct components of alexithymia may follow differential developmental trajectories, underscoring the complexity of age effects [73]. Taken together, the present findings support the view that the relationship between age and alexithymia is not unidirectional, but shaped by developmental, sociocultural, and cohort-related influences. Longitudinal research will be crucial to clarify these dynamics.

Alexithymia has consistently been reported as elevated in IBS populations, with meta-analytic evidence confirming its association with symptom severity and psychosocial impairment [74]. However, age-related patterns within IBS samples remain insufficiently explored. It is plausible that younger individuals with IBS may exhibit higher alexithymic traits due to less consolidated emotion regulation strategies and greater stress reactivity, whereas older patients may benefit from maturational gains in affect regulation and illness coping. Such an interpretation aligns with lifespan models of emotional development and suggests that alexithymia in IBS may not represent a static deficit, but a dynamic vulnerability factor modulated by age-related regulatory processes. Accordingly, our findings encourage a more developmentally informed conceptualisation of alexithymia within IBS, integrating emotional maturation and cohort-related influences into prevailing gut–brain models.

The emerging gap in understanding adds value to the present study, as the relationship between age, Type A behaviour, and demoralization in individuals with IBS has not been adequately explored in previous research. Education was found to be significantly and inversely correlated with DIF and DDF, consistent with both classical and recent studies examining the sociodemographic correlates of alexithymia [75,76].

The second hypothesis focused on the correlations observed among alexithymia, health status, and psychosomatic syndromes. Notably, the significant correlations demonstrated a consistent pattern, with negative associations regarding health status and positive associations concerning psychosomatic syndromes. Specifically, the total score on the TAS-20, as well as the subscales for DIF and DDF, were significantly and inversely related to health status.

Consistent with the existing literature, alexithymia was found to be negatively associated with the health status of patients with IBS [64]. A recent systematic review and meta-analysis by Ismaiel and colleagues [64], considering relevant studies, confirmed the relationship between alexithymia and irritable bowel syndrome. According to the study, Bengtsson and colleagues [77], Portincasa and colleagues [78], Endo and colleagues [79] and Phillips and colleagues [80] detected the relevance of alexithymia for IBS people. However, these studies considered the predictive role of alexithymia as referred to IBS symptom severity or treatment outcomes. Predictive models based on alexithymia represent an added value for research. I all possible cases, interpretation limits should always be considered. Beyond the recognized role of alexithymia in IBS, our results showed an opposite correlation with health status, confirmed by relevant literature contributions [41,81,82,83].

On the other hand, these factors positively correlated with psychosomatic syndromes. According to Bengtsson and colleagues [77], individuals with IBS exhibit significantly higher alexithymia scores compared to patients with Inflammatory Bowel Disease (IBD). Jones and colleagues [84] reported high levels of alexithymia and lower HR-QoL in people with IBS, while Phillips and colleagues detected high alexithymia as a significant predictor of IBS exacerbation [80]. These studies underscore the central role of alexithymia, particularly the total score and the DIF and DDF, for individuals with IBS.

EOT, also traditionally known as operative thinking [26,85,86], can be defined as difficulty in focusing on inner affective dynamics, preferring external stimuli [86]. In our study, it negatively correlated with physical functioning and positively with energy/fatigue and thanatophobia. In line with Panayiotou and colleagues [28,87], EOT was negatively associated with HR-QoL, despite Ismaiel and colleagues [64] reporting its generally low association with somatic diseases. Its relations with thanatophobia appear as new data referring to IBS, despite some studies generally considering the link between alexithymia and phobia [88,89,90]. Unfortunately, the EOT subscale has demonstrated modest reliability, which may constrain the interpretability of our findings.

The second hypothesis explored potential significant correlations between patients’ health status and psychosomatic syndromes. All significant correlations were negative, except for those related to persistent somatization. Thanatophobia and illness denial were found to be negatively correlated with physical functioning. 

Recent research [91] suggested the need for a multidimensional assessment to deepen the relations between illness denial and medical disorders. In line with current and past trends, physical illness is considered to produce illness denial as a defense mechanism (e.g., Silverman and colleagues [92], Fiorentino and colleagues [93], Di Giuseppe [94], Di Giuseppe and Conversano, [95], Silvestro and colleagues [96]). Type A behaviour, irritable mood and demoralization correlated with role limitations due to physical health, and with role limitations due to emotional problems, energy/fatigue, emotional well-being and social functioning. Moreover, irritable mood was associated with pain and general health status, while irritable mood and demoralization were linked to general health status. These results are in line with current psychosomatic research reporting the impact of phenomena on health status and HR-QoL [16,46,56,97,98,99,100,101,102,103].

Regarding the positive and significant correlations, persistent somatization appeared to be associated with role limitations due to physical health, energy/fatigue, social functioning, and pain. However, due to the scarcity of research offering similar results, further interpretation and investigation are needed to explore this positive association more thoroughly.

The third hypothesis addressed the role of alexithymia in health status and psychosomatic syndromes. As noted in previous studies, alexithymia has been recognized as a predictor of poor health status and psychosomatic conditions [64]. In the present study, general levels of alexithymia were found associated with worse physical functioning, higher limitations due to emotional problems, decreased well-being, impaired social functioning, pain, and general health status. Additionally, higher alexithymia scores corresponded to greater disease phobia, thanatophobia, type A behavior, irritable mood, and demoralization. The three alexithymia factors, in combination, were associated with role limitations due to emotional problems and type A behavior. Specifically, DIF was associated with decreased emotional well-being, social functioning, pain, and general health status, while EOT emerged as associated with lower type A behavior, irritable mood, and demoralization. These results confirm the negative role alexithymia in individuals with IBS, despite data related to EOT need to be considered with reference to its internal consistency. Furthermore, it should be noted that while several associations reached statistical significance, some of these should also be interpreted cautiously. In fact, further research is needed to confirm their robustness. Nevertheless, in clinical psychology, even a modest proportion of variance explained may be meaningful and provide important insights due to the multifactorial and probabilistic nature of psychological phenomena.

The final hypothesis, which posited potential gender differences in alexithymia and health-related quality of life, was not supported, as no significant differences were observed between male and female patients. The absence of significant gender effects in our sample is consistent with an updated meta-analytic review indicating only small and heterogeneous gender differences in alexithymia, and with clinical studies in which male and female patients showed similar levels of alexithymia [76,104,105].

Despite these interesting results, this research field presents some issues to be discussed. György Bárdos [105] noted the considerable progress made in understanding the etiological features of IBS, yet highlighted the gap in comprehensive knowledge. According to Bárdos, alexithymia, psychosomatic features, stress, and their relationship with neurophysiological functioning, neuro-gastroenterology, and brain and immune functions are critical fields for advancing diagnosis and treatment.

Nevertheless, the challenges of achieving complete understanding persist. Multidisciplinary approaches remain pivotal in advancing knowledge, though further studies are needed to establish evidence-based, effective diagnostic and intervention protocols. Despite the significance and clinical relevance of the findings, the study has several limitations. The cross-sectional design and non-probabilistic sampling limit the ability to generalize the results through inferential analyses. The predominantly female composition of the sample (81.8%) reflects typical IBS cohorts but may introduce gender-related bias, particularly given established differences in alexithymia. No significant differences were observed between male and female participants, providing some reassurance regarding the robustness of the findings. We also acknowledge that the Cronbach’s alpha for the Externally Oriented Thinking (EOT) subscale was 0.54, below the commonly accepted threshold for research purposes. Accordingly, results involving EOT, especially those derived from regression analyses, should be interpreted with caution.

While patient recruitment and assessment occurred after the national lockdown and under relatively homogeneous clinical conditions, the broader context of the COVID-19 pandemic may have influenced levels of health anxiety, somatization, and quality of life; this potential impact should therefore be considered when interpreting the findings. While our study did not specifically measure the effects of social isolation, this contextual factor may have contributed to heightened health anxiety, somatization, and altered quality of life in the sample. Researchers should consider the potential influence of pandemic-related social stressors when interpreting findings and planning future studies.

Two of the primary instruments employed were self-report measures, reflecting participants’ self-perceptions of alexithymia and health status. Nevertheless, the inclusion of structured clinical interviews and rigorous analytic procedures enhances the validity of the results, which remain consistent with the existing literature and offer novel insights. A further strength of this study is the integration of self-report and clinician-rated assessments. Whereas the TAS-20 captures patients’ subjective experience of alexithymia, the DCPR-SI provides a structured clinician evaluation of psychosomatic syndromes, including alexithymia-related features. Importantly, each patient underwent a thorough clinical interview, ensuring that clinician-rated data accurately reflected their condition. This dual approach affords a more nuanced understanding of the relationship between self-perceived emotional difficulties and clinically observed psychosomatic manifestations, highlighting potential discrepancies between subjective experience and objective assessment. Future research should formally investigate the concordance between these instruments to determine their value for symptom severity and health outcomes in IBS.

## 5. Conclusions

The present study provides important insights into the role of alexithymia in individuals with IBS. Total alexithymia scores, and particularly the Difficulty Identifying Feelings (DIF) and Difficulty Describing Feelings (DDF) subscales, were significantly associated with poorer health status and higher psychosomatic symptom severity, highlighting the centrality of emotional awareness in shaping patient outcomes. An inverse relationship between age and alexithymia was observed, suggesting that younger patients may exhibit greater difficulties in emotional processing, whereas older patients may benefit from maturational gains in affect regulation, emphasising the need for a developmentally informed perspective. While the Externally Oriented Thinking (EOT) subscale demonstrated some associations with physical and emotional outcomes, its low reliability warrants cautious interpretation. The integration of self-report (TAS-20) and clinician-rated (DCPR-SI) assessments provided a nuanced understanding of the interplay between subjective emotional awareness and clinically observed psychosomatic manifestations. Overall, these findings underscore the importance of tailored psychological interventions in IBS, focusing on enhancing emotional awareness and regulation to potentially reduce symptom severity and improve quality of life. Taken together, this study advances understanding of the complex psychobiological mechanisms underlying IBS and highlights the value of multidimensional assessment approaches, while suggesting that future research should examine longitudinal trajectories and the concordance between self-report and clinician-rated measures to inform evidence-based clinical strategies.

## Figures and Tables

**Table 1:** Descriptive statistics for sociodemographic and clinical psychological variables.

Variables	Mean	Standard Deviation
Age	30.90	14.39
Education	14.36	2.86
TAS-20 Total score	55.05	12.76
TAS-20 DIF	18.53	7.11
TAS-20 DDF	14.36	4.01
TAS-20 EOT	25.63	4.61
Physical functioning	17.81	3.55
Role limitations due to physical health	2.60	1.43
Role limitations due to emotional problems	1.34	1.31
Energy/fatigue	8.08	3.81
Emotional well-being	11.80	4.52
Social functioning	5.11	1.71
Pain	5.06	1.94
General health	11.20	3.72
**Variables**	**Frequence**	**Percentage**
Health anxiety	64	44.8%
Disease phobia	10	7.0%
Thanatophobia	11	7.7%
Illness denial	61	42.7%
Persistent somatization	141	98.6%
Functional somatic symptoms secondary to a psychiatric disorder	9	6.3%
Conversion symptoms	33	23.1%
Anniversary reaction	15	10.5%
Irritable mood	21	14.7%
Type A behavior	82	57.3%
Demoralization	78	54.5%
Alexithymia	35	24.5%

**Table 2:** Correlational analyses among sociodemographic, TAS-20, SF-36 and DCPR variables.

Variables	Age	Education
TAS-20 Total score	−0.111	−0.220
TAS-20 DIF	−0.226 **	−0.273 **
TAS-20 DDF	−0.135	−0.207 *
TAS-20 EOT	0.190 *	0.012
Physical functioning	−0.387 **	0.050
Role limitations due to physical health	0.047	0.128
Role limitations due to emotional problems	0.311 **	0.112
Energy/fatigue	0.169 *	−0.037
Emotional well-being	0.248 **	0.142
Social functioning	0.036	0.070
Pain	−0.107	0.082
General health	−0.174 *	−0.051
Health anxiety	−0.013	0.016
Disease phobia	0.023	−0.022
Thanatophobia	0.070	<0.001
Illness denial	−0.005	−0.040
Functional somatic symptoms secondary to a psychiatric disorder	0.063	−0.005
Persistent somatization	−0.029	−0.007
Conversion symptoms	−0.055	−0.071
Anniversary reaction	−0.053	−0.010
Type A behavior	−0.221 **	−0.093
Irritable mood	0.005	0.046
Demoralization	−0.231 **	−0.053
Alexithymia	−0.010	0.020

**Note:** Bold values indicate statistical significance; * <0.05; ** <0.001.

**Table 3:** Correlation analysis among TAS-20 and SF-36 variables.

Variables	TAS-20 Total Score	TAS-20 DIF	TAS-20 DDF	TAS-20 EOT
Physical functioning	−0.199 *	−0.148	−0.175 *	−0.183 *
Role limitations due to physical health	−0.162	−0.184 *	−0.129	−0.037
Role limitations due to emotional problems	−0.236 **	−0.306 **	−0.253 **	0.092
Energy/fatigue	−0.002	−0.056	−0.052	0.169 *
Emotional well-being	−0.258 **	−0.394 **	−0.155	0.087
Social functioning	−0.277 **	−0.383 **	−0.214 *	−0.006
Pain	−0.261 **	−0.306 **	−0.236 **	−0.072
General health	−0.269 **	−0.316 **	−0.165 *	−0.058
Health anxiety	0.165 *	0.106	0.116	0.164
Disease phobia	0.276 **	0.315 **	0.164 *	0.077
Thanatophobia	0.266 **	0.281 **	0.129	0.178 *
Illness denial	0.082	0.149	−0.007	0.035
Functional somatic symptoms secondary to a psychiatric disorder	0.055	0.109	−0.024	0.052
Persistent somatization	0.168 *	−0.144	−0.153	−0.059
Conversion symptoms	0.013	0.127	−0.087	−0.046
Anniversary reaction	−0.026	0.037	−0.094	−0.045
Type A behavior	0.240 **	0.289 **	0.254 **	−0.066
Irritable mood	0.133	0.230 **	0.125	−0.114
Demoralization	0.192 *	0.302 **	0.145	−0.163
Alexithymia	0.204 *	0.137	0.296 **	0.135

**Note:** Bold values indicate statistical significance; * <0.05; ** <0.001.

**Table 4:** Correlation analysis among SF-36 and DCPR variables.

Variables	Physical Functioning	Role Limitations Due to Physical Health	Role Limitations Due to Emotional Problems	Energy/Fatigue	Emotional Well-Being	Social Functioning	Pain	General Health
Health anxiety	−0.036	−0.019	0.051	−0.106	−0.085	0.111	−0.003	−0.147
Disease phobia	−0.072	−0.093	−0.139	−0.098	−0.146	−0.002	−0.054	−0.225 **
Thanatophobia	−0.224 **	−0.088	−0.155	−0.111	−0.235 **	−0.107	−0.136	−0.244 **
Illness denial	−0.202 *	−0.109	0.024	0.078	−0.017	−0.184 *	−0.167 *	−0.118
Functional somatic symptoms secondary to a psychiatric disorder	−0.088	−0.009	−0.079	0.043	0.014	−0.010	−0.018	−0.170 *
Persistent somatization	0.038	0.184 *	0.097	0.180 *	0.128	0.179 *	0.169 *	−0.161
Conversion symptoms	−0.054	−0.103	−0.207 *	0.029	−0.105	−0.063	−0.019	−0.034
Anniversary reaction	0.005	0.047	−0.046	0.060	0.046	0.026	0.024	0.026
Type A behavior	0.043	−0.170 *	−0.409 **	−0.209 *	−0.304 **	−0.219 **	−0.127	−0.110
Irritable mood	−0.117	−0.219 **	−0.344 **	−0.225 **	−0.246 **	−0.333 **	−0.296 **	−0.286 **
Demoralization	0.025	−0.234 **	−0.434 **	−0.324 **	−0.442 **	−0.191 *	−0.062	−0.339 **
Alexithymia	−0.050	0.024	0.005	0.057	−0.008	−0.171 *	−0.027	−0.138

**Note:** Bold values indicate statistical significance; * <0.05; ** <0.001.

**Table 5:** Generalised linear regression analysis among TAS-20 total score (predictor), SF-36 and DCPR variables.

	TAS-20 Total Score		
	B (CI: 95%)	*p*-Value	R^2^
Physical functioning	−0.053 (−0.098/−0.008)	0.023 *	0.036
Role limitations due to physical health	−0.017 (−0.036/0.001)	0.062	0.024
Role limitations due to emotional problems	−0.023 (−0.040/−0.007)	0.006 *	0.051
Energy/fatigue	0.021 (−0.028/0.070)	0.403	0.005
Emotional well-being	−0.065 (−0.122/−0.008)	0.026 *	0.034
Social functioning	−0.043 (−0.064/−0.022)	<0.001 *	0.102
Pain	−0.040 (−0.064/−0.016)	0.001 *	0.062
General health	−0.067 (−0.114/−0.021)	0.005 *	0.053
Health anxiety	0.012 (−0.002/0.027)	0.095	0.019
Disease phobia	0.018 (0.006/0.029)	0.002 *	0.062
Thanatophobia	0.021(0.010/0.033)	<0.000 *	0.081
Illness denial	0.006 (003/0.016)	0.161	0.014
Functional somatic symptoms secondary to a psychiatric disorder	0.006 (−0.007/0.018)	0.368	0.006
Persistent somatization	−0.020 (−0.044/0.005)	0.113	0.017
Conversion symptoms	0.005 (−0.014/0.023)	0.613	0.002
Anniversary reaction	0.000 (−0.013/0.014)	0.968	<0.001
Type A behavior	0.030 (0.005/0.054)	0.016 *	0.039
Irritable mood	0.014 (0.001/0.028)	0.032 *	0.031
Demoralization	0.023 (0.001/0.044)	0.036 *	0.030
Alexithymia	0.023 (0.009/0.037)	0.002 *	0.065

**Note:** Bold values indicate statistical significance; * <0.05.

**Table 6:** Generalised linear regression analysis among TAS-20 factors (predictors), SF-36 and DCPR variables.

	TAS-20 DIF		TAS-20 DDF		TAS-20 EOT		R^2^
	B (CI: 95%)	*p*-Value	B (CI: 95%)	*p*-Value	B (CI: 95%)	*p*-Value	
Physical functioning	−0.047 (−0.144/0.051)	0.346	−0.031 (−0.216/0.155)	0.746	−0.085 (−0.228/0.058)	0.243	0.037
Role limitations due to physical health	−0.034 (−0.074/0.006)	0.093	−0.013 (−0.089/0.062)	0.727	0.012 (−0.046/0.070)	0.684	0.032
Role limitations due to emotional problems	−0.050 (−0.084/−0.016)	0.004 *	−0.077 (−0.141/−0.013)	0.019 *	0.079 (0.030/0.128)	0.002 *	0.161
Energy/fatigue	−0.035 (−0.138/0.068)	0.506	−0.086 (−0.282/0.111)	0.392	0.247 (0.097/0.398)	0.001*	0.069
Emotional well-being	−0.265 (−0.380/−0.150)	<0.001 *	−0.025 (−0.243/0.193)	0.822	0.295 (0.127/0.462)	0.001 *	0.181
Social functioning	−0.099 (−0.143/−0.056)	<0.001 *	−0.026 (−0.109/0.057)	0.543	0.044 (−0.020/0.107)	0.180	0.175
Pain	−0.079 (−0.130/−0.028)	0.003 *	−0.049 (−0.147/0.049)	0.325	0.043 (−0.032/0.118)	0.264	0.106
General health	−0.184 (−0.282/−0.086)	<0.001 *	−0.004 (−0.191/0.184)	0.970	0.091 (−0.053/0.235)	0.215	0.110
Health anxiety	0.004 (−0.028/0.035)	0.818	0.003 (−0.057/0.064)	0.919	0.037 (−0.009/0.084)	0.115	0.027
Disease phobia	0.038 (0.013/0.062)	0.002 *	−0.003 (−0.050/0.043)	0.892	−0.001 (−0.036/0.035)	0.966	0.082
Thanatophobia	0.042 (0.017/0.067)	0.001 *	−0.018 (−0.067/0.030)	0.456	0.020 (−0.017/0.057)	0.283	0.100
Illness denial	0.021 (0.001/0.040)	0.037 *	−0.017 (−0.054/0.020)	0.370	0.002 (−0.027/0.030)	0.897	0.031
Functional somatic symptoms secondary to a psychiatric disorder	0.023 (−0.004/0.049)	0.092	−0.032 (−0.082/0.019)	0.219	0.013 (−0.026/0.052)	0.507	0.024
Persistent somatization	−0.024 (−0.076/0.029)	0.377	−0.038 (−0.137/0.062)	0.460	0.008 (−0.068/0.085)	0.833	0.020
Conversion symptoms	0.058 (0.018/0.097)	0.004 *	0.075 (−0.150/0.001)	0.048 *	−0.011 (−0.068/0.046)	0.703	0.59
Anniversary reaction	0.022 (−0.007/0.051)	0.139	−0.045 (−0.100/0.010)	0.111	0.007 (−0.035/0.049)	0.745	0.022
Type A behavior	0.064 (0.014/0.114)	0.013 *	0.106 (0.010/0.202)	0.030 *	−0.111 (−0.185/−0.035)	0.003 *	0.133
Irritable mood	0.042 (0.014/0.070)	0.003 *	0.018 (−0.035/0.071)	0.510	−0.043 (−0.084/−0.003)	0.036 *	0.095
Demoralization	0.086 (0.044/0.129)	<0.001 *	0.040 (−0.041/0.121)	0.345	−0.119 (−0.181/−0.056)	<0.001 *	0.175
Alexithymia	0.010 (−0.020/0.041)	0.501	0.066 (0.008/0.124)	0.025 *	0.003 (−0.041/0.048)	0.882	0.080

**Note:** Bold values indicate statistical significance; * <0.05.

**Table 7:** Comparisons between male and female patients.

Variables	Males (26)	Females (117)	*p*-Value
TAS-20 Total score	56.34 ± 15.03	54.76 ± 12.25	0.775
TAS-20 DIF	18.34 ± 7.42	18.57 ± 7.07	0.857
TAS-20 DDF	15.11 ± 3.90	14.19 ± 4.03	0.381
TAS-20 EOT	26.69 ± 5.71	25.40 ± 4.33	0.484
Physical functioning	18.92 ± 2.41	17.56 ± 3.71	0.061
Role limitations due to physical health	2.57 ± 1.47	2.60 ± 1.43	0.915
Role limitations due to emotional problems	1.38 ± 1.35	1.34 ± 1.30	0.927
Energy/fatigue	8.42 ± 4.25	8.00 ± 3.73	0.887
Emotional well-being	11.80 ± 5.35	11.80 ± 4.34	0.725
Social functioning	4.84 ± 1.82	5.17 ± 5.17	0.412
Pain	5.03 ± 2.06	5.07 ± 5.07	0.964
General health	11.23 ± 2.53	11.20 ± 11.20	0.776

## Data Availability

The data that supports the findings of this study are available from the corresponding author, E.M.M., upon reasonable request. The data are not publicly available due to privacy and ethical restrictions.

## Abbreviations

The following abbreviations are used in this manuscript:

IBS	Irritable Bowel Syndrome
IBD	Inflammatory Bowel Disease
TAS-20	Toronto Alexithymia Scale
SF-36	36-Items Short Form Survey
DCPR	Diagnostic Criteria for Psychosomatic Research
HR-QoL	Health-Related Quality of life
